# *Pinellia* *pedatisecta* Agglutinin Targets Drug Resistant K562/ADR Leukemia Cells through Binding with Sarcolemmal Membrane Associated Protein and Enhancing Macrophage Phagocytosis

**DOI:** 10.1371/journal.pone.0074363

**Published:** 2013-09-03

**Authors:** Kan Chen, Xinyan Yang, Liqin Wu, Meilan Yu, Xiaoyan Li, Na Li, Shuanghui Wang, Gongchu Li

**Affiliations:** College of Life Sciences, Zhejiang Sci-Tech University, Hangzhou, Zhejiang, China; King's College London, United Kingdom

## Abstract

*Pinellia* *pedatisecta* agglutinin (PPA) has previously been used in labeling fractions of myeloid leukemia cells in our laboratory. We report here that a bacterial expressed recombinant PPA domain b tagged with soluble coxsackie and adenovirus receptor (sCAR-PPAb) preferentially recognized drug resistant cancer cells K562/ADR and H460/5Fu, as compared to their parental cell lines. Pretreatment of K562/ADR cells with sCAR-PPAb significantly enhanced phagocytosis of K562/ADR by macrophages in vivo. Meanwhile, in a K562/ADR xenograft model, intratumoral injection of sCAR-PPAb induced macrophage infiltration and phagocytosis. Furthermore, immunoprecipitation, mass spectrometry and Western blot identified the membrane target of PPA on K562/ADR as sarcolemmal membrane associated protein (SLMAP). An antibody against SLMAP significantly promoted the phagocytosis of K562/ADR by macrophages in vitro. These findings suggest that PPA not only could be developed into a novel agent that can detect drug resistant cancer cells and predict chemotherapy outcome, but also it has potential value in immunotherapy against drug resistant cancer cells through inducing the tumoricidal activity of macrophages.

## Introduction

Resistance to anticancer drugs is a major factor resulting in the failure of chemotherapy. Cancer drug resistance is usually characterized by multiple drug resistance, or multidrug resistance (MDR), a phenomenon whereby cancers resistant to one drug are found to be resistant to other drugs with quite different structures and action modes [1]. The identification of membrane transporters provided the first significant advance in understanding MDR. P-glycoprotein and other ATP-binding cassette (ABC) family members catalyze the efflux of anticancer drugs thereby leading to drug resistance [1,2]. In recent years, a minor population of cancer cells, named cancer stem cells, with the self-renewal capacity, expression of ABC family members, and resistance to apoptosis became a new factor responsible for MDR [3,4,5]. In addition, niche microenvironment hosting cancer cells provides components which lead to insensitivity of cancer cells to anticancer drugs [6,7]. Recently, glycosylation changes have been found to be correlated with MDR [8], providing a new feature for cancer drug resistance.

Analyzing the altered glycosylation of cancer cells in different stages of cancer progression may provide biomarkers for various cancers. In addition to antibodies recognizing specific oligosaccharides, lectins provide an alternative tool for glycosylation analysis [9]. To date, a variety of lectin-based methods have been developed in examining cancer samples. Labeled lectins and lectin microarrays, combined with other technologies such as flow cytometry and proteomic analysis, have been used in identifying biomarkers for various cancers, which include Pancreatic cancer [10], prostate cancer [11], aggressive breast cancer [12,13], ovarian cancer [14], and liver cancer [15]. Cancer stem cells from glioblastoma were reported to be recognized by lectins specific for α-N-acetylgalactosamine, α-N-acetylglucosamine, or galactose [16,17]. Furthermore, lectins including *Maackia* *amurensis* seeds lectin (MASL) [18], Concanavalin A [19], *Polygonatum* *cyrtonema* lectin [20], and various other lectins [21] have been developed into anticancer agents through inducing apoptosis or autophagy. In our laboratory, a mannose specific plant lectin, *Pinellia* *pedatisecta* agglutinin (PPA), has been shown to induce cancer cell death through an adenoviral vector-based gene delivery system, and the methylosome acted as a target for the PPA-mediated cytotoxicity [22]. Collectively, lectins can be utilized in providing diagnosis and prognosis biomarkers, as well as therapeutic agents for a variety of cancers.

We previously determined that PPA recognized fractions of myeloid leukemia cells [23]. However, the characterizations of the PPA recognition were still unknown. Due to that mannose receptor has been found expressed on macrophages [24], we proposed a possible relationship between leukemia cells and the innate immunity. In this work, we found that PPA preferentially recognized drug resistant cancer cells including doxorubicin (ADR) resistant leukemia cells K562/ADR and 5-fluorouracil (5Fu) resistant lung cancer cells H460/5Fu. Treating K562/ADR cells with PPA significantly enhanced phagocytosis of K562/ADR by macrophages in vivo, and induced macrophage infiltration and phagocytosis in a K562/ADR xenograft model. The membrane target of PPA on K562/ADR was determined to be SLMAP.

## Materials and Methods

### Cells

Human chronic myeloid leukemia cell line K562 was purchased from the American Type Culture Collection (Rockville, MD, USA) and maintained in RPMI-1640 medium supplemented with 10% fetal bovine serum, 1% penicillin/streptomycin solution, and 1% L-Glutamine. Human lung cancer cell line H460 was purchased from the American Type Culture Collection and maintained in DMEM supplemented with 10% fetal bovine serum, 1% penicillin/streptomycin solution, and 1% L-Glutamine. Drug resistance cells K562/ADR and H460/5Fu were induced and maintained in our laboratory previously. All cells were cultured at 37°C in 5% CO_2_ humid atmosphere.

### Adenoviral infection

The recombinant serotype 5 adenovirus carrying an enhanced green fluorescent protein gene (Ad-EGFP) was generated in our laboratory previously. Cells were seeded in 24-well plates at 1 x 10^4^/well. For K562 or K562/ADR, cells in each well were treated with 1.6 x 10^9^ viral particles of Ad-EGFP pre-mixed with PBS or 10μg of sCAR-PPAb. For H460 or H460/5Fu, cells in each well were treated with 1.6 x 10^6^ viral particles of Ad-EGFP combined with PBS or 10μg of sCAR-PPAb. After 2 days, cells were examined under a fluorescent microscope (Olympus Corporation, Tokyo, Japan) or a BD FACSAria flow cytometry (BD Biosciences, San Jose, CA, USA).

### Cell viability assay

Cells were plated on 96-well plates at 1×10^4^ per well and treated with ADR, 5Fu, or sCAR-PPAb at concentrations indicated for 48h. The cell viability was determined by a 3-(4,5-Dimethylthiazol-2-yl)-2,5-diphenyltetrazolium bromide (MTT) assay.

### Western blotting analysis

The cell extract or precipitated complexes were subjected to SDS-PAGE and electroblotted onto the nitrocellulose membrane. The membrane was then blocked with TBS-T contaning 5% of BSA at room temperature for 2 hours and incubated with mouse anti-SLMAP antibody (Santa Cruz Biotechnology, Inc., Santa Cruz, CA, USA) or rabbit anti-Actin antibody (Beyotime Institute of Biotechnology, Shanghai, China) overnight at 4°C. The membranes were washed and incubated with appropriate dilution of IRDye 800 donkey anti-mouse IgG or IRDye 700 donkey anti-rabbit IgG (LI-COR, Inc., Lincoln, NA, USA) for 1h at room temperature. After washing with TBS, the membranes were then analyzed using an Odyssey Infrared Imaging System (LI-COR, Inc.).

### Immunoprecipitation and mass spectrometry

K562 or K562/ADR cells at 1 x 10^6^ cells/dish were treated with PBS or 100μg/dish of sCAR-PPAb for 20 minutes. After wash with ice cold PBS, cell lysates were prepared in a lysis buffer (Beyotime Institute of Biotechnology, Shanghai, China). The lysates were then subjected to immunoprecipitation with a goat anti-CAR antibody (Santa Cruz Biotechnology, Inc.) followed by protein A/G conjugated agarose (Santa Cruz Biotechnology, Inc.). Precipitated immunocomplexes were washed 3 times in PBS and boiled in a loading buffer followed by SDS-PAGE and Western blotting analysis. For mass spectrometry, precipitated complexes were subjected to SDS-PAGE followed by silver staining or coomassie brilliant blue staining. Specific bands were excised and digested with trypsin. Peptide mixtures were analyzed under an ABI 4700 MALDI-TOF/TOF mass spectrometer (Applied Biosystems, Framingham, MA, USA). A combined database search was performed using a Mascot software (Version 2.0; Matrix Science, London, UK).

### Ethics statement

All animal studies were approved by the Institutional Animal Care and Use Committee (IACUC) of Hangzhou Normal University, Zhejiang, China.

### In vitro phagocytosis assay

ICR mice 8-10 weeks old were humanely killed, and abdominal cavity macrophages were isolated through wash and collection with PRMT-1640 medium supplemented with 10% fetal bovine serum, 1% penicillin/streptomycin solution, and 1% L-Glutamine. Macrophages were cultured in 6-well plates at 5 x 10^5^ cells/well at 37°C in 5% CO_2_ humid atmosphere overnight. K562/ADR cells were stained with carboxyl fluorescein succinimidyl ester (CFSE), and 1 x 10^6^ cells were mixed with 10μg of a mouse anti-SLMAP mAb (Santa Cruz Biotechnology) or a mouse IgG (Sigma-Aldrich, Co., St. Louis, MO, USA) followed by adding into wells pre-cultured with macrophages. The phagocytosis was observed under a fluorescent microscope (Olympus Corporation) after 2h.

### In vivo phagocytosis assay

ICR mice 8-10 weeks old were injected with 8 x 10^6^ of K562 or K562/ADR stained with CFSE and pretreated with 25μg sCAR-PPAb or the same volume of PBS by peritoneal injections. After 5h, mice were humanely killed, and abdominal cavity cells were collected through wash with PRMT-1640 medium supplemented with 10% fetal bovine serum, 1% penicillin/streptomycin solution, and 1% L-Glutamine. Cells were then stained with a rat anti-F4/80 antibody or normal rat IgG followed by a second staining with a goat anti-rat IgG-PE antibody. The macrophage phagocytic index was analyzed using a BD FACSAria flow cytometer to detect cells showing simultaneous CFSE and PE staining. Antibodies used for flow cytometry were purchased from Santa Cruz Biotechnology.

### Mouse xenograft experiments

Female BALB/c nude mice at 4-5 weeks of age were used for leukemia xenograft. K562/ADR cells were injected subcutaneously into the lower right flank of mice. When the tumors were about 100mm^3^ in size, mice were injected 100μg of sCAR-PPAb or the same volume of PBS intratumorally each day for 7 days. Tumors were harvested at the day after treatment, and F4/80 was analyzed to detect macrophage infiltration by immunohistochemistry. A transmission electron microscope was used to detect macrophage phagocytosis.

## Results

### sCAR-PPAb preferentially recognized drug resistant cancer cells

**Figure 1:**
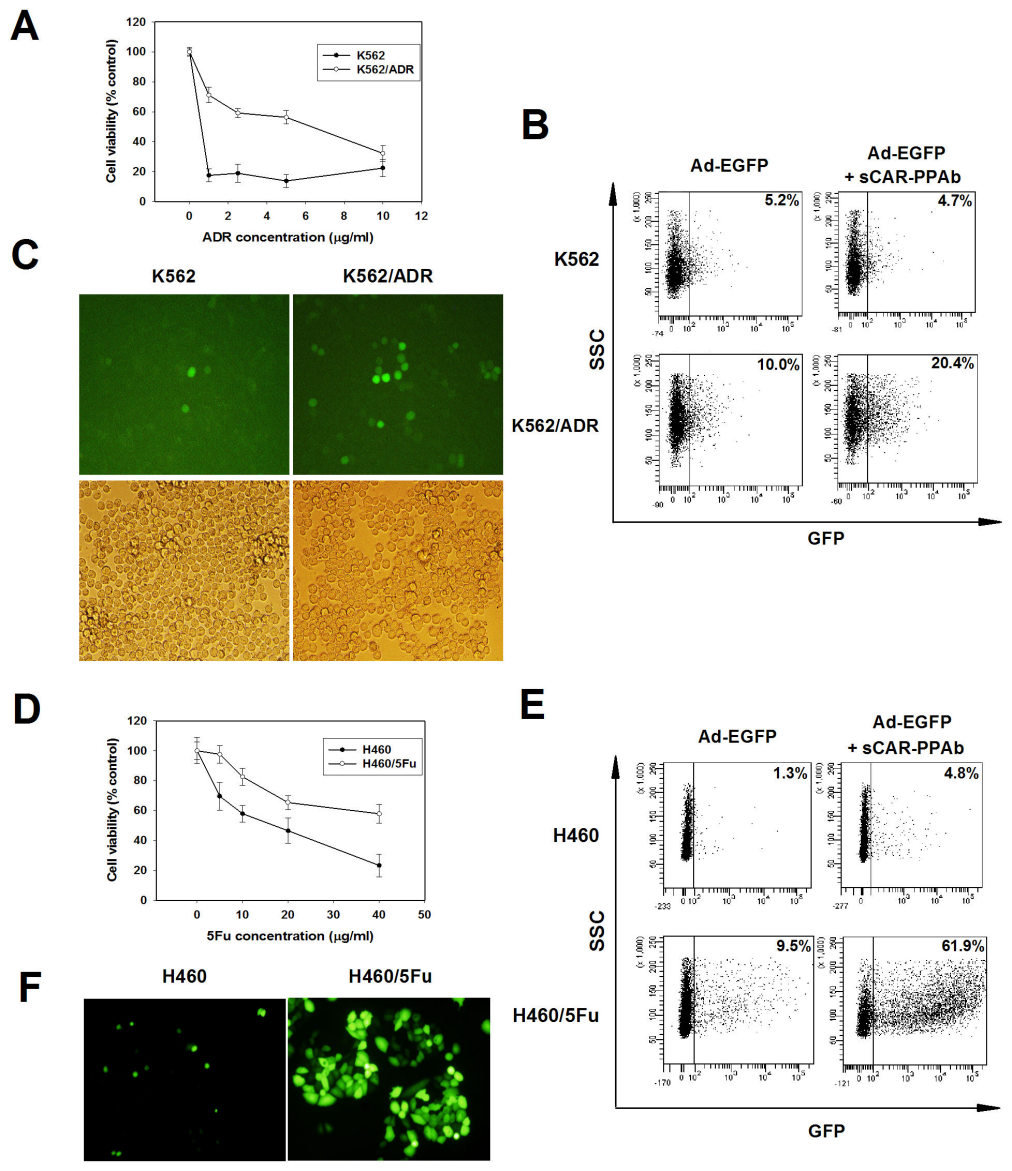
PPA preferentially recognizes drug resistant cancer cells. A. The sensitivity of K562 and K562/ADR to ADR was determined by a MTT assay. B. K562 or K562/ADR cells were treated with sCAR-PPAb combined with Ad-EGFP or PBS for 48h. The portion of EGFP positive cells was analyzed by flow cytometry. Shown is a representative from 3 separate experiments. C. K562 or K562/ADR cells treated with sCAR-PPAb combined with Ad-EGFP for 48h were examined using a fluorescence microscope. D. The sensitivity of H460 and H460/5Fu to 5Fu was determined by a MTT assay. E. H460 or H460/5Fu cells were treated with sCAR-PPAb combined with Ad-EGFP or PBS for 48h. The portion of EGFP positive cells was analyzed by flow cytometry. Shown is a representative from 3 separate experiments. F. H460 or H460/5Fu cells treated with sCAR-PPAb combined with Ad-EGFP for 48h were examined using a fluorescence microscope.

A recombinant fusion protein sCAR-PPAb containing soluble coxsackie adenovirus receptor (sCAR) and the domain b of PPA (D^146^-S^250^) was constructed through a method published previously [23]. The PPA domain b contains two predicted mannose-binding motifs. We first tested whether sCAR-PPAb have higher affinity to doxorubicin (ADR) resistance K562/ADR leukemia cells than the parental K562 cell line. The sensitivity of K562 and K562/ADR cells to doxorubicin was analyzed through MTT assay as shown in Figure 1A. Cells were treated with sCAR-PPAb combined with adenovirus Ad-EGFP followed by fluorescent microscope observation and flow cytometry analysis for portions of enhanced green fluorescent protein (EGFP) positive cells. As analyzed by flow cytometry, sCAR-PPAb did not significantly increase the Ad-EGFP infection in K562 cells. However, the EGFP positive cells were increased from 10.0% to 20.4% upon the sCAR-PPAb treatment (Figure 1B). The higher portion of EGFP positive K562/ADR cells was confirmed by a fluorescent microscope observation (Figure 1C), demonstrating a higher affinity of sCAR-PPAb to K562/ADR cells. To further confirm the recognition of chemoresistant cancer cells by sCAR-PPAb, 5-fluorouracil (5Fu) resistant H460/5Fu cells and the parental H460 cell line were analyzed by sCAR-PPAb and Ad-EGFP. The sensitivity of cells to 5-fluorouracil was determined by MTT assay as shown in Figure 1D. The effect of sCAR-PPAb on the Ad-EGFP infection was analyzed by flow cytometry. Results showed that sCAR-PPAb dramatically increased the infectious rate of Ad-EGFP in H460/5Fu cells, as compared to H460 cells (Figure 1E). The higher portion of EGFP positive cells in H460/5Fu cells was confirmed by a fluorescent microscope observation (Figure 1F). Since previous studies have demonstrated that sCAR alone did not show retargeting effect on adenoviruses [25], our data suggest that PPA is capable to preferentially recognize drug resistant cancer cells.

### Treatment of K562/ADR with sCAR-PPAb enhanced the phagocytosis of K562/ADR by macrophages in vivo

**Figure 2:**
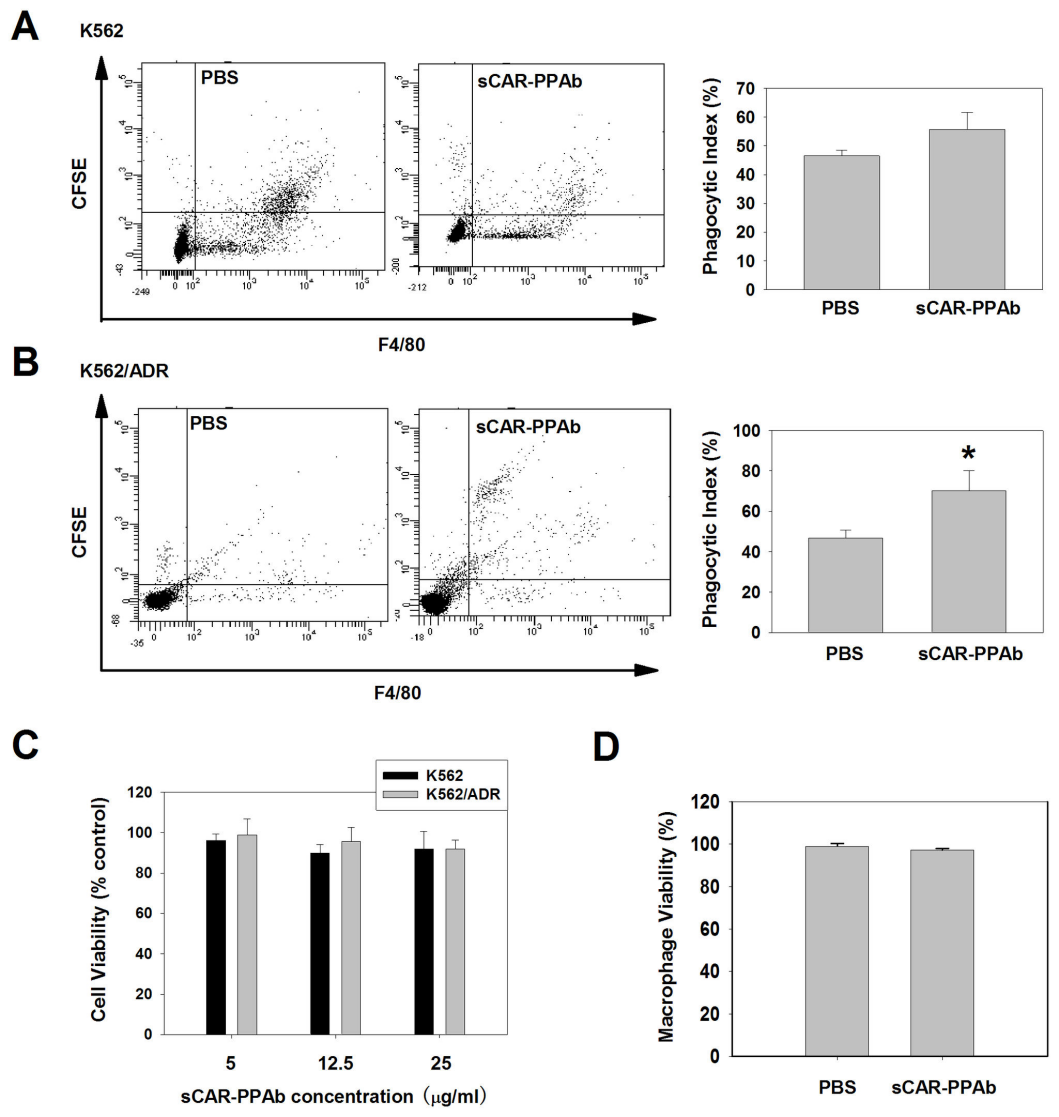
sCAR-PPAb enhances the phagocytosis of K562/ADR cells by macrophages in vivo. A. sCAR-PPAb did not enhance the phagocytosis of K562 cells by macrophages. K562 cells stained with CFSE and pretreated with sCAR-PPAb or PBS were injected into the abdominal cavity in ICR mice. After 5h, cells in the abdominal cavity were then collected and stained with a primary antibody against F4/80 or IgG isotype followed by a goat anti-rat IgG-PE antibody. Cells were then analyzed by flow cytometry. Data were reported as the percent of macrophages undergoing phagocytosis, calculated by CFSE/PE double positive cells divided by all PE positive cells. Values are shown as mean ± SEM (p>0.05, n=3). B. sCAR-PPAb enhanced the phagocytosis of K562/ADR by macrophages. Data were reported as the percent of macrophages undergoing phagocytosis, calculated by CFSE/PE double positive cells divided by all PE positive cells. Values are shown as mean ± SEM (p<0.05, n=3). C. The cytotoxicity of sCAR-PPAb on K562 and K562/ADR was analyzed by MTT assay. D. Mouse macrophages from the abdominal cavity were isolated and cultured in 96-well plates. The cells were then treated with 5μg of sCAR-PPAb for 5 hours. PBS treatment served as the control. The cell viability was analyzed by MTT assay.

Because mannose receptor expression has been shown associated with macrophage functions [24], we then asked whether the binding of sCAR-PPAb to cancer cells can affect the phagocytosis of cancer cells by macrophages. K562 or K562/ADR cells labeled with a green fluorescent dye carboxyl fluorescein succinimidyl ester (CFSE) were pretreated with sCAR-PPAb or PBS followed by intraperitoneal injection into ICR mice. Cells were then collected from the abdominal cavity and analyzed by flow cytometry to determine the portion of macrophages ingesting cancer cells. A F4/80 antibody was used to label mouse macrophages. Cells showing CFSE and F4/80 double positive were considered as the macrophages ingesting cancer cells. As shown in Figure 2A, sCAR-PPAb treatment did not alter the percent of macrophages undergoing phagocytosis for K562 cells. However, upon the treatment of sCAR-PPAb, the percent of macrophages undergoing phagocytosis for K562/ADR cells was significantly increased as compared to the PBS control (Figure 2B). Data indicated that the binding of sCAR-PPAb to K562/ADR enhanced the phagocytosis of K562/ADR by macrophages. In addition, we further demonstrated that sCAR-PPAb did not elicit obvious cytotoxicity to K562 and K562/ADR cells (Figure 2C), as well as mouse macrophages (Figure 2D) by MTT assay, indicating that the enhanced macrophage phagocytosis was not due to an increased level of cell death.

### sCAR-PPAb induced macrophage infiltration and phagocytosis in K562/ADR xenograft

**Figure 3:**
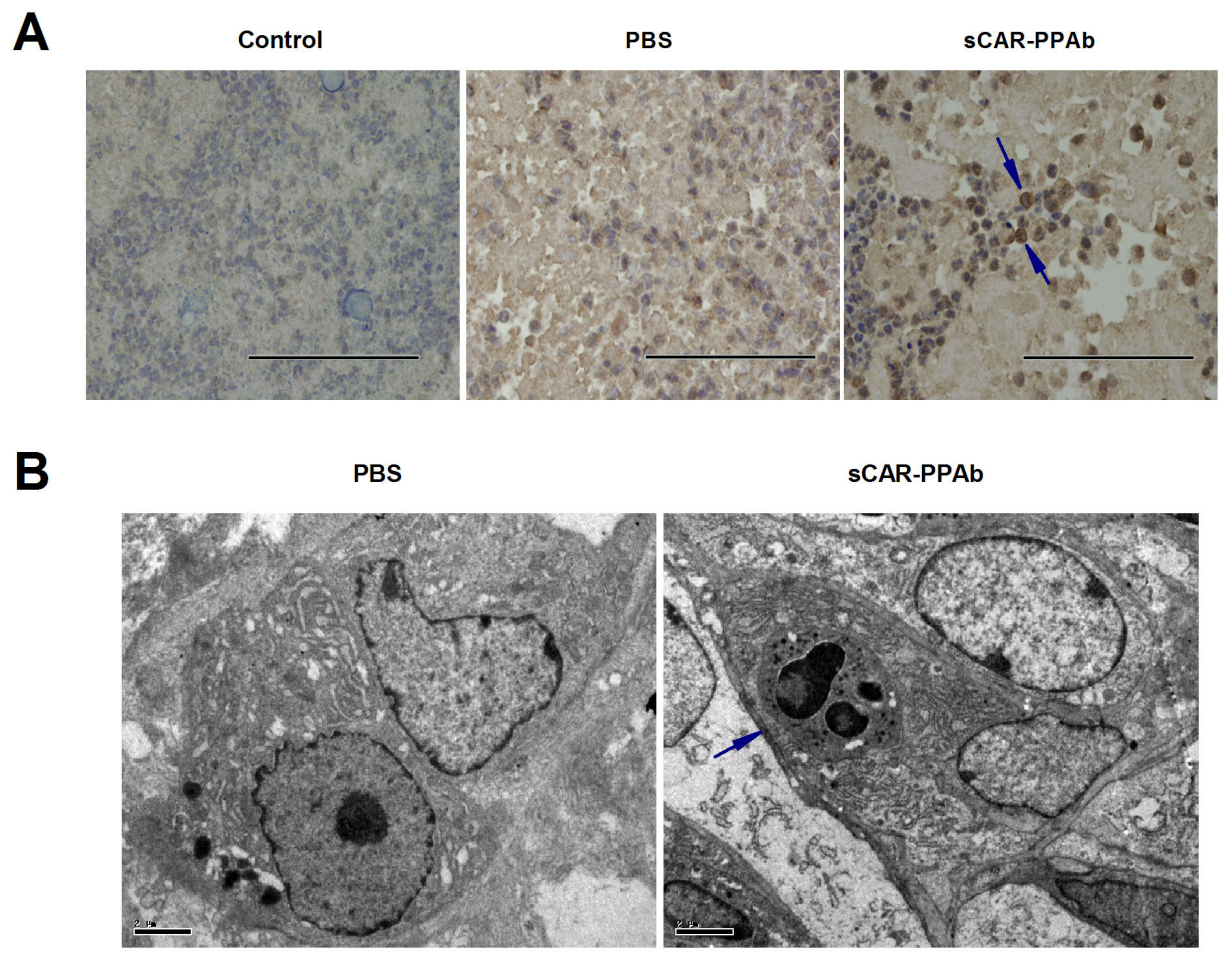
sCAR-PPAb activated macrophages in a K562/ADR xenograft model. BALB/c nude mice were transplanted with K562/ADR cells subcutaneously. sCAR-PPAb were injected intratumorally for 7 days at 100μg/day. PBS injection served as the control. A. Tumors were harvested and F4/80 was analyzed for macrophage infiltration by immunohistochemistry. The control showed IgG isotype staining. Arrows point to cells stained with F4/80. Bars show 200μm (400 x). B. Tumors were analyzed using a transmission electron microscope. The arrow points to cells undergoing phagocytosis. Bars show 2μm.

The effect of sCAR-PPAb on macrophage activation was further investigated through a K562/ADR xenograft model. K562/ADR tumors were established through transplanting cells subcutaneously to BALB/c nude mice. sCAR-PPAb or PBS were injected intratumorally followed by investigating macrophage infiltration by immunohistochemistry, as well as macrophage phagocytosis by transmission electron microscope (TEM) (n=4). Data showed that F4/80 staining specific for mouse macrophages was not detected in all 4 PBS treated tumors (0%). In the sCAR-PPAb group, one mouse-beared tumor disappeared during treatment. Obvious F4/80 staining was detected in two tumors (50%). Figure 3A showed representative F4/80 positive staining in a sCAR-PPAb treated tumor as compared to a PBS treated tumor. TEM analysis further detected obvious phagocytosis in the sCAR-PPAb treated tumors, which was not detected in any PBS treated tumor. The phagocytosis in a sCAR-PPAb treated tumor was shown in Figure 3B. Taken together, our data suggest that sCAR-PPAb treatment induced the macrophage infiltration and subsequent phagocytosis in K562/ADR xenograft tumors.

### Sarcolemmal membrane associated protein (SLMAP) on K562/ADR acted as a target for PPA and macrophages

**Figure 4:**
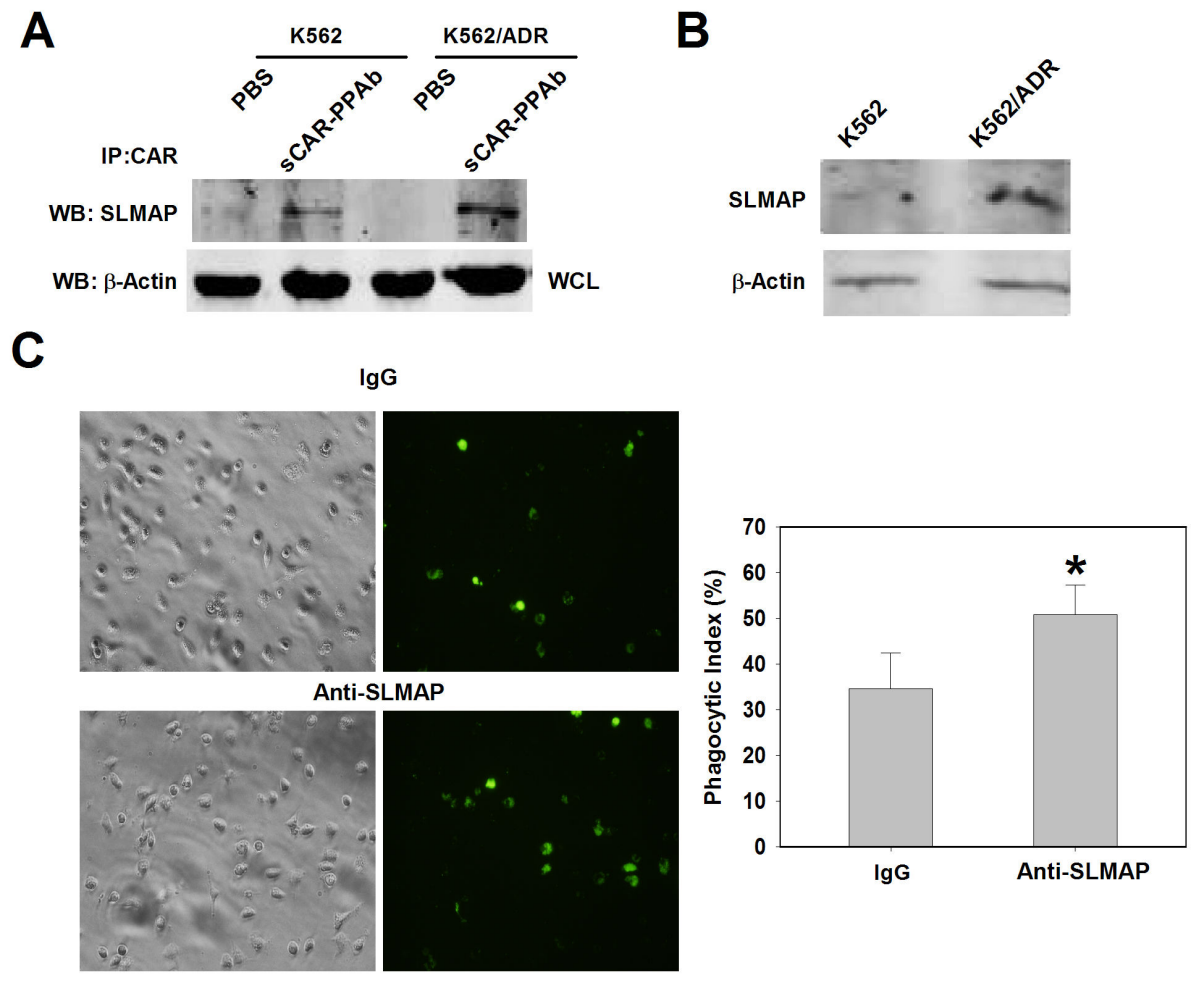
sCAR-PPAb binds with SLMAP. A. sCAR-PPAb combined with a CAR antibody was used in immunoprecipitation. PBS combined with CAR antibody served as the control. Precipitates were analyzed by Western blot for SLMAP. Whole cell lysis (WCL) was monitored for Actin levels. B. SLMAP levels on K562 and K562/ADR were determined by Western blot. Actin served as a loading control. C. A SLMAP antibody enhanced the phagocytosis of K562/ADR by macrophages in vitro. An IgG isotype served as the control. The portion of macrophages ingesting CFSE stained K562/ADR cells was observed under a fluorescence microscope (200 x). A significant enhancement of phagocytosis upon SLMAP antibody treatment was determined (p<0.05).

We then further investigated the target of PPA on K562/ADR cells. The sCAR-PPAb proteins combined with a CAR antibody was used to immunoprecipitate membrane molecules interacting with PPA. Precipitated samples were analyzed by electrophoresis followed by silver staining. A band specifically precipitated by sCAR-PPAb was subjected to mass spectrometry analysis, and was identified as SLMAP (Table 1). To confirm the interaction of PPA with SLMAP, precipitates from both K562 and K562/ADR cells were subjected to Western blot with an antibody against SLMAP. Results showed that a higher level of SLMAP was precipitated by sCAR-PPAb in K562/ADR cells than in K562 cells (Figure 4A). The expression levels of SLMAP in K562/ADR and K562 were examined by Western blot. A significantly higher level of SLMAP was detected in K562/ADR (Figure 4B), confirming the results obtained from the immunprecipitation analysis.

**Table 1:** SLMAP specific peptides identified by mass spectrometry.

**Peptides**	**Calculated mass**	**Observed mass**	**± p.p.m.**	**Start**	**End**	**Sequences**
**1**	1051.6007	1051.4968	-99	497	504	QEIQHLRK
**2**	1210.5811	1210.6388	48	74	84	SSNGTFINSQR
**3**	1266.6378	1266.614	-19	54	63	NHALVWFDHK
**4**	1282.6348	1282.6403	4	766	775	LKFEMTEQEK
**5**	1285.7296	1285.6014	-100	115	126	KVTHGCIVSTIK
**6**	1439.8005	1439.8066	4	21	32	HVYLDEPIKIGR
**7**	1479.7147	1479.7957	55	520	531	CFELQALLEEER
**8**	1493.7086	1493.7345	17	217	229	LEVMGNQLQACSK
**9**	1511.7224	1511.8407	78	442	455	AKESDFSDTLSPSK
**10**	1524.6682	1524.7576	59	37	49	CRPAQNNATFDCK
**11**	1567.8214	1567.7677	-34	753	765	EYEKTQTVLSELK
**12**	1735.8683	1735.9827	66	518	531	QKCFELQALLEEER
**13**	2308.1528	2308.1636	5	484	503	DDLQGAQSEIEAKQEIQHLR
**14**	2462.1794	2462.2078	12	353	373	EQELQAKIEALQADNDFTNER

A protein immunoprecipited by sCAR-PPAb was subjected to trypsin digestion and mass spectrometry. Fourteen peptides were identified and matched to SLMAP.

We then asked whether the expression of SLMAP on K562/ADR was related to macrophage activation. K562/ADR cells labeled with CFSE and pretreated with a SLMAP antibody or IgG isotype were cocultured with macrophages isolated from the peritoneal cavity of ICR mice. K562/ADR cells were then removed and the phagocytic index was examined under a fluorescent microscope. Adherent cells showing green fluorescence were considered as macrophages ingesting K562/ADR cells. As shown in Figure 4C, the SLMAP antibody significantly enhanced the phagocytosis of K562/ADR cells by macrophages, as compared to the IgG control, suggesting a relationship between the SLMAP and macrophage activation.

## Discussion

PPA is a mannose-binding lectin accumulated in the tuber of *Pinellia* *pedatisecta* . In the work presented, a recombinant PPA domain b tagged with sCAR preferentially recognized drug resistant cancer cells including K562/ADR and H460/5Fu cells. The pretreatment of K562/ADR cells with sCAR-PPAb significantly promoted the phagocytosis of K562/ADR by macrophages in vivo. Meanwhile, K562/ADR xenografts injected intratumorally with sCAR-PPAb induced macrophage infiltration and phagocytosis. Furthermore, immunoprecipitation, mass spectrometry and Western blot identified the membrane target of PPA on K562/ADR as SLMAP. A relationship between SLMAP and macrophage was suggested by data from an in vitro phagocytosis analysis.

Altered glycosylation has been reported to be associated with tumor progression [26]. The recognition of drug resistant cancer cells by PPA shown in our work supports the idea that PPA could be utilized in detecting cancer cells resistant to chemotherapies, thereby developed to be a novel agent that can predict chemotherapy outcomes. Second, although glycosylation changes have been linked to drug resistance previously [8], the relationship between the glycosylation changes of drug resistant cancer cells and the immune system remains unknown. In our work, sCAR-PPAb enhanced phagocytosis of K562/ADR cells by macrophages through binding with SLMAP on K562/ADR, indicating an interaction between drug resistant cancer cells and macrophages, as well as a role of SLMAP in mediating the interaction. Previously, CD47 on leukemia stem cells was shown to interact with macrophage signal regulatory protein α (SIRPα) and avoid phagocytosis through inducing a “do not eat” signal [27]. Therapeutic antibodies targeting CD47 interrupted the CD47-SIRPα interaction and enabled phagocytosis of leukemia stem cells by macrophages [28]. In our data, the binding of SLMAP with PPA enhanced phagocytosis by macrophages, suggesting that SLMAP possibly play a role similar to CD47 and suppress phagocytosis through interacting with a cell membrane receptor on macrophages.

SLMAP comprise a family of tail-anchored membrane proterins, and isoforms of SLMAP derived from alternative splicing are targeted to cell membrane, mitochondria, and the microtubule organization centre [29,30,31,32]. Recently, SLMAP was reported to be involved in regulating glucose uptake in type 2 diabetes [33], and modulating intracellular trafficking of hNav1.5 channel in Brugada syndrome [34]. However, to date, SLMAP has not been linked to cancer drug resistance and macrophage phagocytosis. The interaction of SLMAP with PPA may suggest a special glycosylation pattern of SLMAP which provides a recognition signal for PPA. The novel role of SLMAP suggested in our work remains to be confirmed in animal models and clinical analysis.

Monocyte-macrophage lineage is characterized by hallmarks of diversity and plasticity [35]. In response to TLR ligands and IFN-γ, macrophages undergo classical M1 activation and produce IL-12, IL-23, and TNF [36]. Upon IL-4/IL-13 stimulation, macrophages undergo alternative M2 activation and typically express mannose receptor, Fizz1, and chitinase-3 like 3 [37]. In addition, signals such as IL-10 and glucocorticoid hormones induce a macrophage phenotype sharing some properties with M2 cells, called M2-like macrophages [38]. In cancer related inflammation, classical M1-polarized macrophages exhibit antitumor activity and contribute to the T cell mediated elimination and equilibrium phases during tumor progression [39]. Meanwhile, tumor associated macrophages (TAMs) at later stages of tumor progression generally have M2-like phenotype, which is associated with promotion of tissue remodeling and angiogenesis, as well as poor prognosis [40,41]. Therefore, inducing macrophages to skew toward M1 phenotype and elicit tumoricidal activity have become an important therapeutic strategy in cancer treatment. In our work, sCAR-PPAb not only promoted phagocytosis of K562/ADR by macrophages, but also induced macrophage infiltration and phagocytosis in a K562/ADR xenograft model, suggesting that the binding of PPA with K562/ADR may activate macrophage M1 phenotype and enhance the tumoricidal activity of macrophages. The therapeutic effect of PPA on drug resistant cancers remains to be further investigated preclinically and clinically.

We report here that PPA preferentially recognized drug resistant cancer cells. The binding of PPA with K562/ADR cells enhanced macrophage phagocytosis in vivo, as well as macrophage infiltration and phagocytosis in a K562/ADR xenograft model. The target of PPA on K562/ADR was suggested to be SLMAP, which possibly regulated phagocytic signaling in macrophages. Data suggested that PPA could be utilized in detecting drug resistant cancer cells and predicting chemotherapy outcome. Furthermore, a potential therapeutic effect of PPA against drug resistant cancer through activating macrophages was also suggested in our work. Further preclinical and clinical studies are needed to assess the prognosis and therapeutic value of PPA for drug resistant cancers.
